# Factor structure of the diabetes knowledge questionnaire and the assessment of the knowledge of risk factors, causes, complications, and management of diabetes mellitus: A national population-based study in Singapore

**DOI:** 10.1371/journal.pone.0272745

**Published:** 2022-08-10

**Authors:** Kumarasan Roystonn, P. V. AshaRani, Fiona Devi Siva Kumar, Peizhi Wang, Edimansyah Abdin, Chee Fang Sum, Eng Sing Lee, Siow Ann Chong, Mythily Subramaniam

**Affiliations:** 1 Research Division, Institute of Mental Health, Singapore, Singapore; 2 Clinical Research Unit, Diabetes Centre, Admiralty Medical Centre, Singapore, Singapore; 3 Clinical Research Unit, National Healthcare Group Polyclinics, Singapore, Singapore; 4 Lee Kong Chian School of Medicine, Nanyang Technological University, Singapore, Singapore; 5 Saw Swee Hock School of Public Health and Department of Medicine, National University of Singapore, Singapore, Singapore; Government College University Faisalabad, Pakistan, PAKISTAN

## Abstract

This study evaluated the knowledge of diabetes mellitus and predictors of the level of diabetes knowledge among the general public of Singapore. Confirmatory factor analysis and exploratory factor analysis were used to evaluate the fit of different factor models for the diabetes knowledge questionnaire. Multiple linear regressions were performed to determine the sociodemographic characteristics associated with diabetes knowledge. The final factor model identified three domains for diabetes knowledge: general knowledge, diabetes specific knowledge and causes of diabetes, and complications of untreated diabetes. Overall knowledge scores were 23.8 ± 2.4 for general diabetes knowledge, 2.3 ± 0.8 for diabetes specific knowledge, 2.3 ± 1.2 for causes, and 5.2 ± 1.2 for complications of untreated diabetes. Patients with diabetes were more knowledgeable than adults without diabetes in the population. While the general public in Singapore has adequate knowledge of diabetes, misconceptions were identified in both groups which underscores the need to tailor specific educational initiatives to reduce these diabetes knowledge gaps.

## Introduction

Diabetes mellitus is a complex and chronic disease associated with a state of chronic high blood glucose level, or hyperglycaemia. Diabetes comprises mainly two types, Type 1(insulin dependent) and Type 2 (non-insulin dependent) [1]. Type 2 diabetes affects more than 400 million people around the world. By 2040, it is projected that there will be more than 640 million people with diabetes worldwide [2–4]. To date, the International Diabetes Federation has estimated that Asia accounts for 60% of the world’s population with diabetes, with more than 50% of persons with type 2 diabetes being undiagnosed [4]. In Singapore, as in other countries in Asia, diabetes is a major public health concern [5]. In 2017, diabetes was the seventh leading cause of morbidity and premature mortality in Singapore [6].

The development of Type 2 diabetes involves multiple factors and mechanistic pathways, notably epigenetics, defective insulin activity, glucotoxicity, lipotoxicity, inflammation, oxidative stress, and pancreatic β-cell dysfunction [6–11]. Environmental and lifestyle, as well as genetic factors, can increase the risk of diabetes. Lifestyle factors including diet quality and quantity, weight, and physical activity (i.e., excessive calorie intake, high fat diets, and increased sedentary lifestyles) can lead to obesity and insulin resistance [12, 13]. The well-recognised symptoms of diabetes are polyuria, polydipsia and polyphagia [14]. Other symptoms include tiredness, recurrent infections, slow-healing wounds, blurred vision and gastrointestinal complications. Diabetes can further result in damage to various organs including the eyes, heart and blood vessels, and kidneys, leading to diabetic neuropathy, blindness, heart diseases and renal disorders [15]. Although it is well established that individuals can improve their disease outcomes and reduce the risk of complications by taking precautionary measures such as lifestyle modifications [16], and regular monitoring of blood glucose levels (e.g., Haemoglobin A1c; HbA1c) [17, 18], many people become aware that they have diabetes only after complications such as vision loss and renal complications manifest [19]. Early awareness of diabetes risk thus provides an opportunity to introduce preventive interventions to stop or delay the disease onset [20, 21].

Knowledge, attitude and practice (KAP) studies on diabetes worldwide have increasingly demonstrated the importance for greater awareness of diabetes symptoms, risk factors, suitable lifestyle practices and regular monitoring of blood glucose levels [22–25]. There are several studies which have examined the knowledge of diabetes among Asian populations [25–27]. However, these studies were often conducted in small, community or clinic-based samples and focused mainly on patients diagnosed with diabetes [26–29]. To date, there have been several other studies in Asia which have evaluated diabetes related knowledge among adults with diabetes and those without diabetes [30–33]. Yet, to our best knowledge, no other research has thoroughly examined the current level of knowledge of diabetes in a national population-based study in Singapore. Therefore, the current study aimed to assess the level of diabetes related knowledge among adults with diabetes and those without diabetes in the general population and evaluate the predictors associated with diabetes knowledge in Singapore.

## Materials and methods

### Sample and procedures

The present cross-sectional study is part of a larger, national population-based KAP study of diabetes among Singapore residents [34]. The sample was randomly selected via a disproportionate stratified sampling design from a national database of Singapore citizens and permanent residents (aged 18 years and above). In addition, certain minority sub-populations (i.e., Malay and Indian ethnic groups, and those aged 65 years and above) were oversampled to improve the reliability of the parameter estimates for these groups.

The randomly selected residents were sent notification letters followed by home visits by a trained interviewer from a survey research company to obtain their informed consent to participate in the study. For residents who agreed to participate, face-to-face interviews were conducted in their preferred language (English, Mandarin, Malay, or Tamil). Responses were captured using computer-assisted personal interviewing. Individuals who could not be contacted due to incomplete or incorrect addresses, living outside of the country, institutionalization, or hospitalization at the time of the survey, as well as, individuals who were incapable of participating due to language barriers or severe physical or mental conditions were excluded from the study. The study commenced in February 2019 but was temporarily suspended from March 2020 –July 2020 due to the lockdown phase in response to the Coronavirus pandemic in Singapore. The study resumed in August 2020 and was completed in September 2020, achieving a sample size of 2895 and a study response rate of 66.2%. Written informed consent was obtained from all participants prior to the survey and all study procedures. The study protocol and the study questionnaire were approved by the ethics committee, National Healthcare Group Domain Specific Review Board (DSRB No. 2018/00463).

### Diabetes knowledge questionnaire

The diabetes knowledge questionnaire was developed based on literature review and validated by a panel of healthcare professionals who were experts in diabetes care and treatment [34]. Pretesting of the questionnaire was performed to evaluate the questionnaire’s readability, clarity, acceptability and consistency among the population by ensuring a good representation across age, gender, ethnicity and education of the sample. The questionnaire was also translated and tested in Mandarin, Malay, and Tamil. The diabetes knowledge questionnaire of 29 items, included questions on general diabetes knowledge, causes of diabetes, and likely complications of untreated diabetes. Sociodemographic information such as age, gender, ethnicity, education, marital status, monthly personal income, and employment status were also collected.

### Statistical analysis

All analyses were conducted with Stata version 15 and Mplus version 8.2. Weighted means and standard deviations are presented for continuous variables, while frequencies and weighted percentages are displayed for categorical variables. To ensure representativeness of the data to the general population, the survey sample was weighted by age and ethnicity to account for the complex survey design.

#### Factor analysis of the diabetes knowledge questionnaire

A series of exploratory factor analyses (EFA) and confirmatory factor analyses (CFA) were conducted with the diabetes knowledge questionnaire. In Mplus, the CFA was first estimated and tested to evaluate the factor structure of the questionnaire. As there were items on the questionnaire measured on an ordinal or binary scale, a weighted-least-squares with a mean-adjusted and variance-adjusted (WLSMV) estimator was used to model the observed polychoric/tetrachoric correlation matrix (the categorical option) with a pairwise deletion of missing data. However, due to the poor fit of the initial CFA model, subsequent analyses were performed with approximately two split-half samples (n = 1447; n = 1448) randomly generated from the study sample.

Using the WLSMV estimator in the factor analysis, pairwise deletion of missing data and an oblique geomin rotation were conducted to explore the dimensionality of the first half-sample (n = 1447). The following criteria were utilized to determine the number of factors in the EFA: (i) eigenvalues > 1 (ii) visual inspection of scree plot, (iii) identification of satisfactory factor loadings on each factor (i.e., loadings >0.3, no cross-loadings), and (iv) the robustness of interpretability for each solution. During each analysis, the factor loading of the questionnaire items were explored. Each rotated solution was examined in order to identify and remove items based on the following ranked criteria: (i) consistently low loadings of <0.3 across all factor models, (ii) consistently cross-loading across all models, (iii) lowest loading, and (iv) cross-loading.

Derived factors from the EFA were then validated using CFA in the second half-sample (n = 1448). A WLSMV estimator was applied to examine the underlying polychoric correlation matrix. The following fit indices were utilized to compare the overall fit of the models and their complexities: (i) root mean square error of approximation (RMSEA), (ii) comparative fit index (CFI), (iii) Tucker-Lewis index (TLI). Both the CFI and TLI values range from 0 to 1, with higher values representing better fit; CFI values above 0.95 and TLI values above 0.90 were considered to be of excellent fit [35]. With regards to the RMSEA, values below 0.08 indicate moderate fit, while values of 0.05 or less indicate close fit to the observed data [36]. Standardized root mean squared residual values (SRMR) were also evaluated, which indicate acceptable fit when values are smaller than 0.08 and excellent fit when values are smaller than 0.05 [35, 36]. Internal consistency of each scale was evaluated using the composite reliability values for the best fitting model for the full sample, where the acceptable level was set at 0.70 or greater [37]. Multiple linear regressions were conducted within the full sample to examine the sociodemographic correlates (i.e., age, gender, ethnicity, education, marital status, employment, personal monthly income, and diabetes diagnosis) of each factor.

## Results

Sociodemographic characteristics and the respective weighted percentages of the sample are reported in Table 1. Of the 2895 participants, 823 (29.9%) were aged 21–34 years; 1474 (51.6%) were female; 796 (75.8%) were Chinese; 1860 (61.7%) were married or cohabiting; and 637 (20.4%) had primary level education and below. Also, 436 (9.1%) were diagnosed with diabetes and 2459 (90.9%) were not diagnosed with diabetes in this study.

**Table 1:** Sociodemographic characteristics of the sample (n = 2895).

	N (sample)	Weighted %
Age group		
21–34	823	29.9%
35–49	719	28.2%
50–64	774	26.8%
65 and above	579	15.1%
Gender		
Female	1,474	51.6%
Male	1,421	48.5%
Ethnicity		
Chinese	796	75.8%
Malay	974	12.7%
Indian	918	8.6%
Others	207	2.9%
Education		
Primary and below	637	20.4%
Secondary School	684	20.3%
Pre-University/Junior College	126	4.8%
Vocational Institute/ITE	267	6.6%
Diploma	479	18.5%
Degree, Professional Certification and above	702	29.5%
Marital status		
Married/cohabiting	1,860	61.7%
Single	731	29.2%
Divorced/separated	154	5.0%
Widowed	149	4.1%
Employment		
Employed	1,933	70.5%
Economically inactive^a^	829	25.4%
Unemployed	133	4.1%
Monthly personal income (SGD)		
Below 2,000	1,455	45.3%
2,000 to 3,999	698	23.9%
4,000 to 5,999	318	12.8%
6,000 to 9,999	183	7.8%
10,000 & above	117	5.7%
Undisclosed	124	4.5%
Diabetes diagnosis		
No diabetes	2459	90.9%
Has diabetes	436	9.1%

Frequencies and percentages may not tally to 100% due to missing data.

^a^Economically inactive includes retired, homemaker, student, and the physically disabled.

### Factor structure of the diabetes knowledge questionnaire

An initial CFA was conducted on the 29-item diabetes knowledge questionnaire within the full sample, utilizing a four first-order factor structure. However, this indicated a poor fit to the data (WLSMV χ^2^ = 1685.75, RMSEA = 0.03, CFI = 0.73, TLI = 0.70, SRMR = 0.11). Descriptive information of all 29 items of the diabetes knowledge questionnaire can be found in S1 Appendix. An inspection of the initial EFA results, the correlation matrix, as well as the conceptual similarities among respective items in the half-sample (n = 1447) revealed that the questionnaire conformed well to a three-factor model. The three-factor model was then utilized for subsequent analyses. A series of EFAs were conducted to examine the underlying factor structure of each of the domains.

For Domain A, the plot of eigenvalues of the initial 10 items indicated that either a one-factor or two-factor solution was plausible. Upon examining the rotated factor models, four items were removed due to a consistently low loading of < 0.3 and cross-loadings. This led to a single factor solution of six items for the general knowledge (GK) scale, which was found to be optimal. A CFA of this six-item unidimensional model resulted in an acceptable fit (WLSMVχ^2^(9) = 40.78; RMSEA = 0.05; CFI = 0.96; TLI = 0.93; SRMR = 0.03). A total score was calculated by summing all items, with higher scores indicating higher knowledge. The composite reliability value for GK was acceptable at 0.71.

For Domain B, eigenvalues for the underlying correlation matrix indicated that a one-factor to three-factor solution was plausible. After an examination of the rotated factor solutions, two items were removed due to consistently low loadings of <0.3 and cross-loadings. A two-factor solution comprising diabetes specific knowledge (DK) and knowledge of causes of diabetes (CK) was found to be optimal. A CFA of the two-factor solution indicated an acceptable fit: (WLSMVχ^2^(13) = 24.34, RMSEA = 0.03, CFI = 0.94, TLI = 0.9, SRMR = 0.06). Scores on the domain were generated by summing the correct responses on the respective items, with higher scores indicating higher knowledge. The composite reliability of DK and CK was poor, at 0.50 and 0.66 respectively.

For Domain C, the plot of eigenvalues for the underlying correlation matrix suggested a one-factor to three-factor solution. However, upon inspection of the EFA solutions, four items were removed due to consistently low loadings of <0.3 and cross-loadings, and a unidimensional structure for complications of untreated diabetes (CPK) was found to be most optimal. The CFA of this six-item unidimensional model indicated an acceptable fit: (WLSMVχ^2^(9) = 24.14, RMSEA = 0.03, CFI = 0.96, TLI = 0.94, SRMR = 0.06). A score was calculated by summing the number of correct responses of all items on the CPK scale, with higher scores indicating higher knowledge. The composite reliability of CPK was high at 0.83.

The statistical fit of the final models and domains are presented in Table 2. The final 19-item questionnaire consists of three knowledge domains: Domain A, a single factor model consisting of six items on the general knowledge of diabetes (GK), measured on a five-point Likert scale ranging from strongly agree to strongly disagree; Domain B, a two-factor model with binary response options of correct and incorrect, consisting of a 3-item sub-scale on diabetes specific knowledge (DK) and a 4-item sub-scale on the causes of diabetes (CK); and Domain C, a single factor model consisting of six items on the complications of untreated diabetes (CPK) measured on binary response options of correct and incorrect.

**Table 2:** Fit statistics of the final CFA models for each domain of the diabetes knowledge questionnaire (19 items).

Final model (Domain A)			
Fit statistics of CFA model		Item description	Standardized Factor Loading
WLSMV χ^2^ (df 9)	40.78, p < 0.001	**General knowledge of diabetes (GK)**	
RMSEA	0.049	Diabetes can be prevented.	0.446
CFI	0.959	Diabetes is treatable.	0.452
TLI	0.932	Lipid (e.g., Cholesterol) and blood pressure control is necessary in diabetic patients.	0.519
SRMR	0.026	Achieving your ideal weight helps control diabetes.	0.703
		High fibre foods (e.g., wholegrain, oatmeal, broccoli etc) help to keep blood sugar levels steady.	0.573
		If untreated, diabetes can reduce a person’s life-expectancy (an average time a person is expected to live, based on their current age and other demographic factors including gender).	0.521
Final model (Domain B)			
Fit statistics of CFA model		Item description	Standardized Factor Loading
WLSMV χ^2^ (df 13)	24.338, p = 0.028	**Diabetes specific knowledge (DK)**	
RMSEA	0.025	A fasting blood sugar level of 13millimoles per litre (>200miligrams/ 100millilitres) is too high	0.509
CFI	0.938	There are two main types of diabetes: Type 1 (insulin-dependent) and Type 2 (non-insulin dependent).	0.618
TLI	0.9	Lack of insulin in blood	0.362
SRMR	0.06	**Causes of diabetes (CK)**	
		Eating less sugar	0.456
		High blood pressure	0.561
		Mental stress	0.548
		Underweight	0.701
		Correlation coefficient between two latent factors	-0.298
Final model (Domain C)			
Fit statistics of CFA model		Item description	Standardized Factor Loading
WLSMV χ^2^ (df 9)	23.14, p = 0.006	**Complications of untreated diabetes (CPK)**	
RMSEA	0.033	Kidney damage / Kidney failure	0.699
CFI	0.964	Heart failure	0.847
TLI	0.94	Stroke	0.802
SRMR	0.063	Loss of feeling in the hands, fingers and feet	0.591
		Cuts and other minor injuries heal more slowly	0.499
		Oral health problems	0.547

All standardized factor loadings were significant at *p* < 0.001

### Sociodemographic determinants of diabetes knowledge

Table 3 presents the weighted percentages of the responses on the diabetes knowledge questionnaire. Overall, mean (± SD) knowledge scores on the respective sub-scales were 23.8 ± 2.4 (out of 30) for GK, 2.3 ± 0.8 (out of 3) for DK, 2.3 ± 1.2 (out of 4) for CK, and 5.2 ± 1.2 (out of 6) for CPK. Almost all (98.1%) of the participants knew that cuts and other minor injuries heal more slowly in persons with diabetes. The majority (92.0%) of participants were also aware that kidney damage or kidney failure were likely complications of untreated diabetes. Most participants thought that high blood pressure (66.3%) and mental stress (54.9%) cause diabetes (Table 3).

**Table 3:** Weighted percentages of responses on the diabetes knowledge questionnaire (19 items).

**General knowledge of diabetes (GK)**												
	Strongly Agree		Agree		Neither		Disagree		Strongly Disagree		Don’t Know	
	n	%	n	%	n	%	n	%	n	%	n	%
1. Diabetes can be prevented.	651	20.5%	1,854	65.7%	219	8.3%	149	5.0%	11	0.5%	11	0.1%
2. Diabetes is treatable.	461	12.8%	1,997	66.9%	231	9.3%	177	9.7%	22	1.2%	7	0.2%
3. Lipid (e.g., Cholesterol) and blood pressure control is necessary in diabetic patients.	582	16.3%	2,000	70.0%	189	8.2%	83	3.8%	5	0.1%	36	1.6%
4. Achieving your ideal weight helps control diabetes.	588	17.2%	1,904	65.4%	204	8.7%	164	7.0%	11	0.5%	24	1.1%
5. High fibre foods (e.g., wholegrain, oatmeal, broccoli etc) help to keep blood sugar levels steady.	551	16.3%	1,984	68.4%	224	8.8%	71	3.8%	6	0.1%	59	2.6%
6. If untreated, diabetes can reduce a person’s life-expectancy (an average time a person is expected to live, based on their current age and other demographic factors including gender).	678	21.6%	1,930	70.7%	140	3.4%	115	3.7%	23	0.5%	9	0.2%
**Diabetes specific knowledge (DK)**												
	Incorrect				Correct				Don’t Know			
	n		weighted %		n		weighted %		n		weighted %	
1. A fasting blood sugar level of 13millimoles per litre (>200miligrams/ 100millilitres) is too high	603		24.8%		1,339		38.2%		953		37.0%	
2. There are two main types of diabetes: Type 1 (insulin-dependent) and Type 2 (non-insulin dependent).	353		13.7%		2,201		71.3%		341		15.1%	
3. Lack of insulin in blood (likely causes diabetes)	317		10.8%		2,326		80.6%		252		8.6%	
**Causes of diabetes (CK)**												
Please indicate the likely causes of diabetes:	Incorrect				Correct				Don’t Know			
	n		weighted %		n		n		weighted %		n	
1. Eating less sugar	542		18.1%		2,343		81.5%		10		0.4%	
2. High blood pressure	1,821		63.5%		970		33.7%		104		2.8%	
3. Mental stress	1,622		51.5%		1,178		45.1%		95		3.4%	
4. Underweight	1,073		32.8%		1,743		64.7%		79		2.6%	
**Complications of untreated diabetes (CPK)**												
Please indicate the likely complications of untreated diabetes:	Incorrect				Correct				Don’t Know			
	n		weighted %		n		n		weighted %		n	
1. Kidney damage / Kidney failure	164		6.2%		2,675		92.0%		56		1.8%	
2. Heart failure	533		20.9%		2,263		75.9%		99		3.2%	
3. Stroke	556		22.5%		2,256		74.9%		83		2.7%	
4. Loss of feeling in the hands, fingers and feet	287		13.0%		2,535		84.4%		73		2.6%	
5. Cuts and other minor injuries heal more slowly	66		1.7%		2,816		98.1%		13		0.2%	
6. Oral health problems	342		11.9%		2,425		83.4%		128		4.7%	

**Fig 1:**
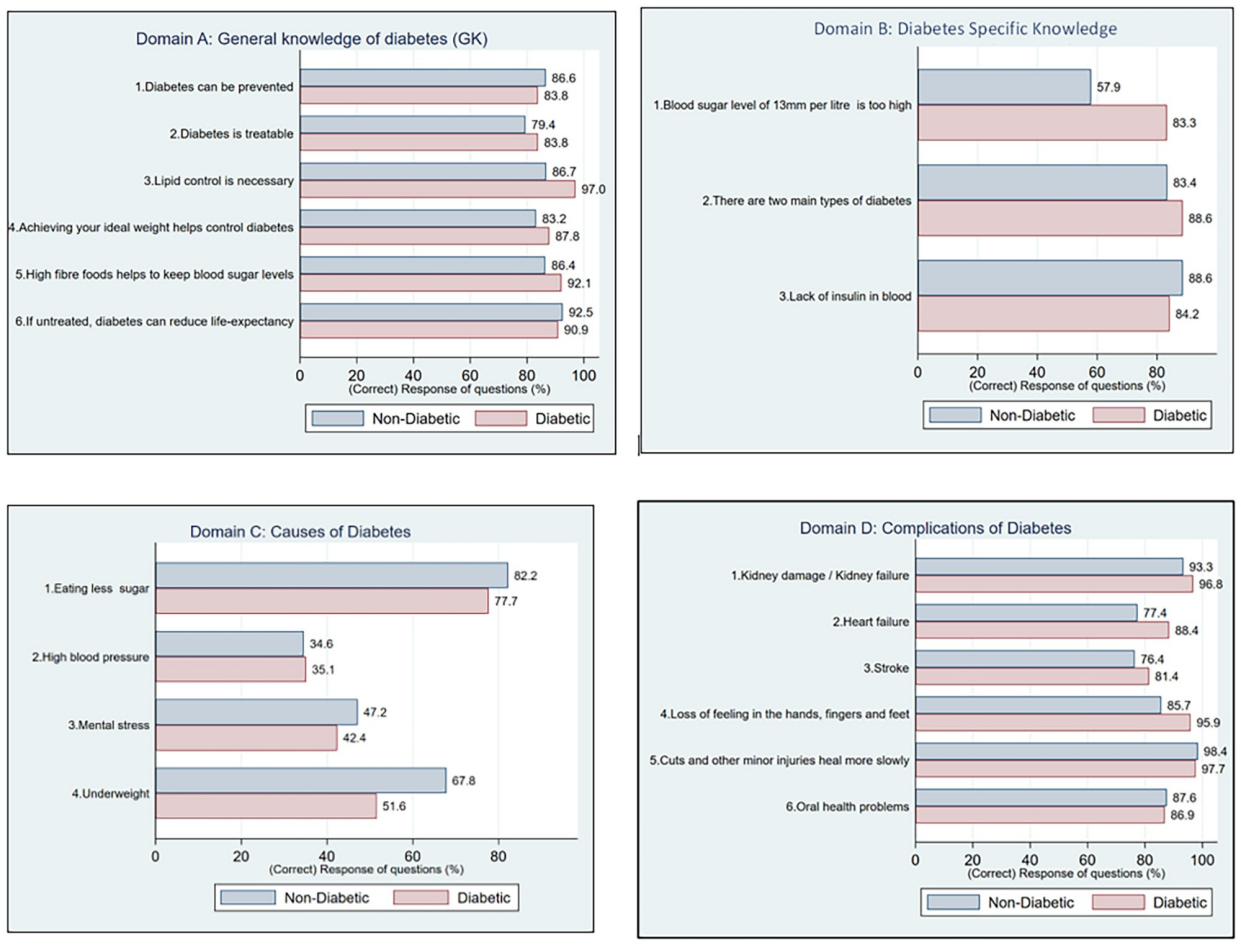
Assessment of diabetes knowledge among participants with diabetes and those without diabetes.

From Fig 1, both the participants with diabetes (90.9%), and those without diabetes (92.5%), were aware that if left untreated, diabetes can reduce one’s life expectancy. Participants without diabetes (42.1%) were not aware of high blood sugar levels, while 83.3% of participants with diabetes knew that a blood sugar level of 13 millimoles per litre is too high. Of the 2459 participants without diabetes, 16.6% were not aware that there are two main types of diabetes (Type 1 and Type 2), compared to 88.6% of participants with diabetes who knew this (Fig 1). While participants with no diabetes (86.6%) and those diagnosed with diabetes (83.8%) both knew that diabetes can be prevented, about 13.3–16.8% of the participants without diabetes were unaware that high fibre food, and having good weight and lipid control helps to control diabetes (Fig 1).

Results of the multiple linear regression analyses are presented in Table 4. After accounting for listwise deletion of missing data, respective cases in the multiple linear regression model for GK was n = 2677, DK was n = 1786, CK was n = 2586, and CPK was n = 2532.

**Table 4:** Results of the multiple linear regression examining correlates of diabetes knowledge.

	General knowledge of diabetes(GK) ^a^				Diabetes specific knowledge(DK) ^b^				Causes of Diabetes(CK) ^c^				Complications of untreated diabetes(CPK) ^d^			
	β	95% CI		p	β	95% CI		p	β	95% CI		p	β	95% CI		p
		Lower	Upper			Lower	Upper			Lower	Upper			Lower	Upper	
Age																
18 to 34	ref				ref				ref				ref			
35 to 49	0.17	-0.25	0.58	0.44	-0.02	-0.19	0.15	0.80	0.08	-0.12	0.28	0.44	-0.12	-0.35	0.10	0.29
50 to 64	0.11	-0.37	0.60	0.65	0.08	-0.11	0.28	0.39	0.07	-0.17	0.31	0.56	-0.06	-0.32	0.20	0.66
65 and above	0.27	-0.29	0.83	0.35	0.09	-0.14	0.33	0.44	0.16	-0.12	0.45	0.26	0.15	-0.12	0.42	0.28
Gender																
Female	ref				ref				ref				ref			
Male	-0.10	-0.38	0.17	0.47	-0.10	-0.22	0.02	0.09	-0.01	-0.15	0.12	0.85	-0.19	-0.33	-0.05	0.01
Ethnicity																
Chinese	ref				ref				ref				ref			
Malay	0.83	0.58	1.09	0.00	0.18	0.06	0.29	0.00	-0.13	-0.26	0.00	0.06	0.16	0.03	0.30	0.02
Indian	0.91	0.66	1.15	0.00	0.26	0.16	0.35	0.00	-0.22	-0.34	-0.10	0.00	0.14	0.02	0.27	0.03
Others	0.45	-0.06	0.96	0.08	0.11	-0.08	0.30	0.25	-0.13	-0.34	0.08	0.24	0.43	0.24	0.62	0.00
Education																
Degree, professional certification, and above	ref				ref				ref				ref			
Primary and below	-0.77	-1.28	-0.26	0.00	-0.20	-0.44	0.04	0.11	-0.54	-0.82	-0.26	0.00	0.18	-0.08	0.45	0.18
Secondary	-0.78	-1.25	-0.32	0.00	-0.23	-0.44	-0.03	0.03	-0.25	-0.48	-0.02	0.03	0.03	-0.21	0.27	0.80
Pre-University/Junior College	-0.51	-1.35	0.33	0.23	0.16	-0.07	0.38	0.17	-0.08	-0.40	0.23	0.60	0.25	-0.07	0.58	0.12
Vocational training	-0.61	-1.19	-0.03	0.04	-0.26	-0.50	-0.03	0.03	-0.08	-0.36	0.20	0.57	-0.28	-0.64	0.08	0.12
Diploma	-0.11	-0.54	0.31	0.60	0.05	-0.12	0.21	0.60	-0.10	-0.29	0.10	0.33	0.06	-0.16	0.27	0.60
Marital Status																
Married/Cohabiting	ref				ref				ref				ref			
Single	-0.62	-1.03	-0.21	0.00	-0.21	-0.37	-0.05	0.01	0.21	0.02	0.40	0.03	-0.33	-0.55	-0.11	0.00
Divorced/Separated/ Widowed	-0.19	-0.63	0.25	0.41	-0.11	-0.29	0.08	0.27	-0.09	-0.34	0.15	0.45	-0.05	-0.27	0.17	0.65
Employment																
Employed	ref				ref				ref				ref			
Economically inactive	-0.16	-0.50	0.17	0.33	0.02	-0.14	0.18	0.79	-0.02	-0.21	0.17	0.86	-0.04	-0.23	0.14	0.63
Unemployed	-0.91	-1.63	-0.19	0.01	-0.24	-0.53	0.05	0.10	0.05	-0.31	0.40	0.80	0.03	-0.37	0.43	0.89
Monthly Personal Income (SGD)																
Below 2,000 or no income	ref				ref				ref				ref			
2,000–3,999	-0.05	-0.41	0.30	0.76	-0.02	-0.19	0.14	0.78	-0.20	-0.39	-0.02	0.03	-0.01	-0.20	0.18	0.91
4,000–5,999	0.19	-0.29	0.68	0.44	-0.04	-0.26	0.18	0.72	0.05	-0.19	0.30	0.66	-0.09	-0.34	0.15	0.45
6,000–9,999	-0.17	-0.81	0.47	0.60	-0.26	-0.52	0.00	0.05	-0.06	-0.36	0.24	0.68	-0.23	-0.56	0.09	0.16
10,000 and above	0.26	-0.46	0.98	0.48	0.11	-0.18	0.39	0.46	-0.06	-0.43	0.32	0.77	0.22	-0.15	0.60	0.24
Diabetes Diagnosis																
No Diabetes	ref				ref				ref				ref			
Has Diabetes	-0.03	-0.42	0.36	0.89	0.22	0.06	0.38	0.01	-0.11	-0.35	0.14	0.39	0.12	-0.08	0.32	0.25

β–Unstandardized regression coefficient; 95% CI– 95% confidence interval of β

^a^After accounting for listwise deletion of missing data, cases in multiple linear regression model: 2677. Mean: 23.8 ± 2.4

^b^After accounting for listwise deletion of missing data, cases in multiple linear regression model: 1786. Mean 2.3 ± 0.8

^c^After accounting for listwise deletion of missing data, cases in multiple linear regression model: 2586. Mean 2.3 ± 1.2

^d^After accounting for listwise deletion of missing data, cases in multiple linear regression model: 2532. Mean 5.2 ± 1.2

The multiple linear regression analyses revealed significantly higher GK and DK scores among ethnic minorities, i.e., Malays [GK (β = 0.83, p< 0.01); DK (β = 0.18, p< 0.01)] and Indians [GK (β = 0.91, p< 0.01); DK (β = 0.26, p< 0.01)] as compared to the Chinese. DK was additionally found to be higher among those with diabetes (β = 0.22, p = 0.01) compared to those without diabetes. CK was particularly high among those who were single (β = 0.21, p = 0.03) while CPK scores were significantly higher among ethnic Malays (β = 0.16, p = 0.02), Indians (β = 0.14, p = 0.03), and Others (β = 0.43, p< 0.01) (vs. Chinese). GK scores were negatively associated with being single (β = -0.62, p< 0.01), unemployed (β = -0.91, p = 0.01), or having lower levels of education (primary or lower (β = -0.77, p< 0.01), secondary (β = -0.78, p< 0.01), vocational training (β = -0.61, p = 0.04) vs. degree and above).

DK scores were significantly lower among those who were single (β = -0.21, p = 0.01), had a higher personal income ($6,000 - $9,999) (β = -0.26, p = 0.05), and secondary education (β = -0.23, p = 0.03) or vocational training (β = -0.26, p = 0.03). Also, CK scores were significantly lower among Indians (β = -0.22, p< 0.01), those with lower personal income ($2,000 - $3,999) (β = -0.20, p = 0.03), and those with secondary education (β = -0.25, p = 0.03) or primary education and below (β = -0.54, p< 0.01). Also, males (β = -0.19, p = 0.01), and being single (β = -0.33, p< 0.01) were significantly associated with lower scores for CPK.

## Discussion

This study aimed to examine the general public’s level of knowledge of diabetes among individuals diagnosed with diabetes and those without diabetes in Singapore. In this study, participants’ knowledge was assessed based on their understanding of diabetes, which included the likely causes, risk factors, symptoms, and complications of diabetes. Overall, despite a lack of awareness in certain aspects, this study found that there was adequate knowledge of diabetes among adults with no diabetes and those with diabetes in the whole population.

This finding is in line with other studies [32, 38–40], which reported better scores on diabetes related knowledge among those with diabetes compared to individuals with no diabetes. These patients could have received diabetes health education during their interactions with the healthcare system. Thus, an encouraging explanation of these high scores among persons with diabetes could be that they reflect the quality of diabetes education received at the diabetes clinics where patients attend regularly.

One possible reason for the knowledge deficiencies observed in the current study may be attributed to misconceptions surrounding certain issues like the risk factors and preventative measures related to diabetes. In this study, Singaporeans were generally able to identify the symptoms and complications of diabetes, though they were not as well versed in the risk factors that may lead to the disease. Majority of the participants in this study, believed that high blood pressure and mental stress are likely causes of diabetes, which are one of the most common misconceptions reported in other population studies as well [32, 41].

Interestingly, while more than 80% of the general population in the current study knew that diabetes and its complications could be prevented, individuals with no diabetes did not know that it can be managed or prevented through lifestyle measures such as high fibre foods, lipid control, and good weight control. These findings are similar to a study conducted in India [42], and also with studies elsewhere [43–45]. The study with the Indian adult population revealed that a majority (82%) believed diabetes was not preventable by altering lifestyle practices and less than a third of them knew that diet and weight were important components of effective diabetes management [42].

This current study also revealed that a significant proportion of individuals without diabetes did not know there are different types of diabetes, and were not as aware of abnormal blood glucose levels. The findings remain consistent with a previous study conducted in Singapore [38], and could be attributed to a lack of personal interest, access, and exposure to the information regarding diabetes.

Research has revealed that poor self-management is a significant barrier to effective prevention or management of diabetes complications [46]. Participation in preventative care strategies such as self-monitoring of blood glucose levels have been shown to reduce the incidence and progression of the disease [47]. It is necessary for health care services to know what people think about a disease and its prevention and management, as misconceptions act as a formidable barrier for the management and prevention of a disease. It is clear that if prevention is to be effective, diabetes education needs to address these gaps in knowledge with more rigour. Other research have demonstrated positive results in altering misconceptions through education for example, regarding risk factors and self-monitoring of blood glucose levels [47, 48]. In addition, healthcare services at various levels should become more aware of the need to screen for, and educate individuals with inadequate knowledge of diabetes [49].

This study revealed a relationship between income levels and diabetes knowledge. Other reports are in agreement with our results, that is, lower income levels were associated with poorer diabetes knowledge [26, 29–31]. Of all the significant predictors of diabetes knowledge, education was the only modifiable risk factor in this study. Consistent with other research [27–33], higher education levels were associated with higher levels of diabetes knowledge in this study. One possible explanation is that those of higher academic levels (and hence, higher income levels) are more able to obtain knowledge from various media sources. In addition, they may have fewer communication barriers with health care professionals, and a better ability of comprehending information. Expectedly, those with little or no formal education were observed to be the least knowledgeable across diabetes knowledge domains in this study.

The current study found that ethnic minority groups (Indians, Malays, and Others) were significantly more knowledgeable about symptoms and complications, insulin deficiency, and abnormal blood glucose levels when compared to Chinese Singaporeans. Our results differ from a few other studies [30, 32]. One plausible explanation could be that the ethnic minorities such as Indians and Malays, are more susceptible to the development of diabetes and its complications than the Chinese [50]. As such, they could have been exposed to diabetes health education delivered as part of their regular interactions with the healthcare system, or they may have acquired the information through close contacts with a history of diabetes. Consequently, the diabetes knowledge gap among the Chinese must be addressed with culturally-tailored diabetes education.

The study has some limitations. Individuals who were institutionalised, hospitalised or uncontactable during the study period, as well as those with language difficulties were excluded from the study. Hence, the results may have been underestimated or overestimated. Moreover, the cross-sectional nature of the study does not allow for causal relationships to be established. Nonetheless, the current study has its strengths in that it was a nationwide population-based study with a representative public sample, ensuring high quality of data and generalizability of the findings. The factor analyses revealed a marked stability and robust factor model for the diabetes knowledge questionnaire in the study. This study has provided more precise and valuable data for the purposes of policy-making, development of diabetes literacy and health promotion programs, as well as for future research.

## Conclusions

The level of knowledge of diabetes in persons with diabetes and persons without diabetes was found to be adequate, except in one situation where both groups thought that high blood pressure and mental stress cause diabetes. Individuals without diabetes also did not know about the levels of blood glucose that were considered abnormal compared to patients with diabetes. These misconceptions can be effectively addressed through suitable diabetes health education. Knowledge regarding diabetes can vary greatly depending on one’s education, ethnicity and socioeconomic status. Understanding these variables will be important in designing prevention and management strategies for diabetes. This study reinforces the view that the main approach to managing diabetes effectively is to improve understanding and management of the disease by means of suitable widespread educational campaigns.

## Supporting information

S1 Appendix Descriptive statistics of the initial 29 items of the diabetes knowledge questionnaire. (PDF)
