# Supplementary material for: Factor structure of the diabetes knowledge questionnaire and the assessment of the knowledge of risk factors, causes, complications, and management of diabetes mellitus: A national population-based study in Singapore
Source: PLoS One. 2022 Aug 10;17(8):e0272745. doi: 10.1371/journal.pone.0272745 (PMC9365176; doi:10.1371/journal.pone.0272745)
Supplement: S1 Appendix — (PDF) [file pone.0272745.s001.pdf]

Questions on diabetes knowledge	Strongly Agree (5)		Agree (4)		Neither (3)		Disagree (2)		Strongly Disagree (1)		Don't Know / Refused†	
	n	weighted %	n	weighted %	n	weighted %	n	weighted %	n	weighted %	n	weighted %
Diabetes is an infectious disease*	42	1.1%	83	2.0%	62	1.5%	1,373	47.7%	1,328	47.6%	7	0.1%
If diabetes is not treated, the amount of sugar in the blood usually increases.*	789	20.7%	1,749	62.4%	166	7.9%	130	6.1%	24	1.1%	37	1.7%
If I am diabetic, my children have a higher chance of being diabetic.*	439	12.7%	1,818	65.2%	312	9.7%	276	11.0%	30	1.0%	20	0.5%
Diabetes can be prevented.	651	20.5%	1,854	65.7%	219	8.3%	149	5.0%	11	0.5%	11	0.1%
Diabetes is treatable.	461	12.8%	1,997	66.9%	231	9.3%	177	9.7%	22	1.2%	7	0.2%
Lipid (e.g., Cholesterol) and blood pressure control is necessary in diabetic patients.	582	16.3%	2,000	70.0%	189	8.2%	83	3.8%	5	0.1%	36	1.6%
Achieving your ideal weight helps control diabetes.	588	17.2%	1,904	65.4%	204	8.7%	164	7.0%	11	0.5%	24	1.1%
High fibre foods (e.g., wholegrain, oatmeal, broccoli etc) help to keep blood sugar levels steady.	551	16.3%	1,984	68.4%	224	8.8%	71	3.8%	6	0.1%	59	2.6%
Fresh fruits can be eaten freely with little effect on blood sugar levels.*	137	3.1%	952	26.8%	417	13.5%	1,241	50.5%	117	5.3%	31	0.8%
If untreated, diabetes can reduce a person's life-expectancy (an average time a person is expected to live, based on their current age and other demographic factors including gender).	678	21.6%	1,930	70.7%	140	3.4%	115	3.7%	23	0.5%	9	0.2%

**Questions on diabetes knowledge**	**Incorrect (0)**		**Correct (1)**		**Don’t Know / Refused†**	
	n	weighted %	n	weighted %	n	weighted %
A fasting blood sugar level of 13millimoles per litre (>200miligrams/ 100millilitres) is too high	603	24.8%	1,339	38.2%	953	37.0%
There are two main types of diabetes: Type 1 (insulin-dependent) and Type 2 (non-insulin dependent).	353	13.7%	2,201	71.3%	341	15.1%

**Questions on the likely causes of diabetes**	**Incorrect (0)**		**Correct (1)**		**Don’t Know / Refused†**	
	n	weighted %	n	n	weighted %	n
Eating less sugar	542	18.1%	2,343	81.5%	10	0.4%
High blood pressure	1,821	63.5%	970	33.7%	104	2.8%
Lack of physical activity*	453	15.1%	2,408	83.8%	34	1.1%
Mental stress	1,622	51.5%	1,178	45.1%	95	3.4%
Underweight*	1,073	32.8%	1,743	64.7%	79	2.6%
Lack of insulin in blood	317	10.8%	2,326	80.6%	252	8.6%
Failure of the body to use insulin*	284	8.6%	2,330	82.6%	281	8.9%

**Questions on the likely complications of untreated diabetes**	**Incorrect (0)**		**Correct (1)**		**Don’t Know / Refused†**	
	n	weighted %	n	n	weighted %	n
Foot ulcer*	289	11.0%	2,509	85.6%	97	3.4%
Poor vision*	126	5.1%	2,748	93.9%	21	1.1%
Loss of vision*	247	10.8%	2,599	87.3%	49	1.9%
Kidney damage / Kidney failure	164	6.2%	2,675	92.0%	56	1.8%
Heart failure	533	20.9%	2,263	75.9%	99	3.2%
Stroke	556	22.5%	2,256	74.9%	83	2.7%
Loss of feeling in the hands, fingers and feet	287	13.0%	2,535	84.4%	73	2.6%

Questions on the likely complications of untreated diabetes (continued)	Incorrect (0)		Correct (1)		Don’t Know / Refused†	
	n	weighted %	n	n	weighted %	n
Foot ulcer*	289	11.0%	2,509	85.6%	97	3.4%
Cuts and other minor injuries heal more slowly	66	1.7%	2,816	98.1%	13	0.2%
Amputation*	66	1.9%	2,804	97.2%	25	0.9%
Oral health problems	342	11.9%	2,425	83.4%	128	4.7%

† Refused/Missing responses were treated as missing data.
* indicates items which were removed.
